# 

**Published:** 1894-12-08

**Authors:** 


**Notices of Births, Deaths, and Marriages** are inserted in this column at a charge of 2s. 6d. for an announcement not exceeding four lines.

**Notice to Advertisers and Subscribers.**

All Advertisements, Orders for Copies of The Hospital, and Business Communications should be addressed to **The Publisher, NOT TO THE EDITOR,** The Hospital, 428, Strand, W.C.

The Cost of Subscription, post free, is as follows:—Per week, 2½d.; per quarter, 2s. 8d.; per half-year, 5s. 3d.; per annum, 10s. 6d. Foreign:—Per Annum, 12s. 8d.; payable, in all cases, in advance.

**NOTICE TO CORRESPONDENTS.**

All MS., Letters, Books for Review, and other matters intended for the Editor should be addressed The Editor, The Lodge, Porchester Square, London, W.

The Editor cannot undertake to return rejected MS., even when accompanied by stamped directed envelope.

**"Burdett's Hospital Annual," 1894.**

Fifth year of publication. 900 pp. Boards, gilt lettering. A most complete guide to British, Colonial, Indian, and American Hospitals, Dispensaries, Nursing and Convalescent Institutions, and Asylums. Price 5s. Published by The Scientific Press (Limited), 428, Strand, W.C.

**NOTICE.—The Index for Vol. XVI. (ending September 24th, 1894) is on sale at the Office of this Journal, Price 2½d., post free.**

### SCRAPS AND GLEANINGS.

The Queen has sent a gift of old linen to King's College Hospital.

A cheque of £50 has been received at the Salford Royal Infirmary from an anonymous donor.

Some dramatic entertainments are being undertaken by the inhabitants of Stirling in aid of the Royal Local Infirmary.

The Lord Mayor of London has accepted the presidency of the Hospital Saturday Fund Association during his year of office.

Gifts of clothing, &c., are very acceptable, we are reminded, for the little inmates of the Bristol Hospital for Sick Children.

A jumble sale in aid of the Reigate Benefit Nursing Association was held last week in the Drill Hall, Reigate, which proved a great success, towards the financial support of the association.

A new infectious diseases hospital has been opened at Ashby-de-la-Zouch, the total cost of the work has been £640, and the building is adapted in every way for the purposes for which it is intended.

It is gratifying to see that the Edinburgh Royal Infirmary has so well reduced its expenses, without interfering with the efficient working of the institution. Last year the cost per bed was £63 13s., this year it was only £58 10s.

Within the last few days a service was held at St. Michael's Church, Liverpool, in aid of the fund of the Royal Southern Hospital. The Lord Mayor attended in state, and collections to the value of £100 were made during the ceremony.

The usual grant from the Hospital Sunday Fund has been received at the Hospital for Consumption, Brompton, viz., £1,437 10s., an increase on the amount for last year. Mrs. Elizabeth Allan has left £5,000 duty free to this institution.

The annual report of the Northern Counties Hospital for Incurables is of a highly satisfactory nature, in so far as the general administration of the home goes. Subscriptions show some falling off, but various substantial legacies have been received.

The 149th anniversary meeting of the subscribers of the Salop Infirmary has just been held, when, in accordance with a very old custom, a procession was formed and proceeded to St. Chad's Church, where a special service was held and a collection made in aid of the funds of the institution.

Last week handsome testimonials, on behalf of numerous subscribers, were presented to Mr. Robert Allan and Mr. Frederick Brown, secretary and treasurer respectively, of the Douglas Hospital, Isle of Man, these testimonials being in recognition of the valuable services of the two gentlemen.

At the first meeting of the newly-constituted committee of the Aberdeen City Hospital Committee, various improvements in the internal arrangements of the institution were discussed. The old pavilions, it was further shown, could be extended to accommodate eight additional beds, at a cost of £130.

The committee of management of the Metropolitan Hospital, Kingsland Road, have arranged for the reception of Jewish patients as well as for the treatment of out-patients. A "Kosher" kitchen has been provided, and every facility will be given to Jews to observe their religious rites and ceremonies.

The Victoria Hospital for Consumption and Diseases of the Chest, Craiglaith House, was inaugurated yesterday by Lord Stormonth-Darling. The movement which has resulted in the founding of this hospital started in the year of the Queen's Jubilee, when premises were taken in Bank Street.

One special feature in the latest report has been pointed out in connection with the Hull Homœopathic Dispensary, which is the great percentage of the people who are receiving the benefits of the institution being aged and infirm, out of 108 such beneficiaries 31 had been over 50 years of age and 28 over 60.

A good deal of sensation has been caused in Stranraer by the intimation of the resignation of Drs. Easton, Munroe, and Cluckie from their position of surgeons to the institution. The interests of the Cottage Hospital, it is asserted, are hereby at stake, and it is much to be hoped the resignations may be withdrawn.

The annual meeting of the friends and supporters of the Children's Hospital, Upper Temple Street, Dublin, has just taken place. No less an expenditure than £600 has had to be incurred during the period under review on alterations, drainage, &c. Further accommodation is much needed for chronic and convalescent patients in the institution.

The results of the grand bazaar held last week in the Municipal Buildings at Southport in favour of the funds of the new local infirmary has realised all expectations as to its success. The total amount received was £5,389, the special object of the scheme being to raise money for furnishing the new building and laying out the spacious ground.

The question of providing additional accommodation for infectious disease patients is occupying the attention of the Sanitary Committee of the City Council of Coventry. The present fever hospital while sufficient for ordinary purposes is inadequate under pressure of serious epidemics. It is further suggested to erect a small-pox hospital outside the town.

The most pleasing portion of the Tynemouth Victoria Jubilee Infirmary's annual report is that the pence of the working men of the borough has reached the sum of £400, or one-half of the total amount subscribed. Through this increased co-operation of the working classes it is considered probable the infirmary will soon extend its sphere of usefulness.

A special meeting of the governors of the Richmond Royal Hospital was lately called. The Duke of Teck presided. The resolution at the meeting ran thus: "To authorise the completion of the executive portion of the new buildings as provided in the original scheme for the building of the Princess May ward for children and the other enlargement of the hospital.

Further isolation hospital accommodation is to be supplied for the county of Wilts. A meeting on this head was held lately at Trowbridge, when it was moved that the local Town Council "will be prepared to form a hospital committee in one of the manners directed by the Isolation Hospital Acts; and further, that they are prepared to borrow money required for the structural expenses of the same."

### Books Received.

Alexander Gardner.
"Housing the People." By Sir Hugh Gilzean Reid, J.P.

Methuen and Co.
"The Vaccination Question." By Arthur W. Hutton, M.A.

Charles Griffin and Co.
"A Manual of Ambulance." By J. Scott Riddell.

John Wright and Co., and Simpkin, Marshall, and Co.
"Diseases of the Upper Respiratory Tract." By P. Watson Williams, M.D.

Cassell and Co.
"Elements of Surgical Pathology." By A. J. Pepper, F.R.C.S.

Smith, Elder, and Co.
"Artificial Feeding and Food." By W. B. Cheadle, F.R.C.P.

John Bale and Sons.
"Diseases of the Breast." By W. Roger Williams, F.R.C.S.

**Periodicals and Pamphlets.**—English Illustrated Magazine, Review of Reviews, Sunday at Home, Girls' Own Paper, Boys' Own Paper, Leisure Hour, Penny Tales, Revue Générale des Sciences, Maandblad voor Ziekenverpleying, The Pherapist, Indian Me ico-Chirurgical Review, Child's Guardian, Charity Organisation Review, London, Forward, Journal of State Medicine, Some Arguments against Vaccination, Medical Pioneer, Zeitschrift für Krankenpflege, The Tri-State Medical Journal, Tit-Bits, The Strand Magazine, The Strand Novelette, and The Million.

### The Student of Medicine.

### APPOINTMENTS.

A. G. Andrews, F.R.C.S., reappointed Junior Anæsthetist to the Manchester Royal Infirmary. W. A. Bond, M.A., M.D., B.C., D.P.H.Camb., M.R.C.P.Lond., appointed Medical Officer of Health for Southwark. J. B. Byles, M.R.C.S.Eng., L.R.C.P. Lond., D.P.H.Camb., appointed House Surgeon to Addenbrooke's Hospital, Cambridge. W. H. C. Davey, M.R.C.S., L.R.C.P. Lond., appointed Honorary Assistant Surgeon to the Liverpool Infirmary for Children. R. T. Howlett, M.D.Lond., appointed Assistant Bacteriologist in Dr. Macfadyen's Department at the British Institute of Preventive Medicine. G. Hudson, M.B. Edin., appointed House Surgeon to the Huntingdon County Hospital. J. S. Hudson, L.R.C.P., M.R.C.S., appointed House Surgeon to the East London Hospital for Children. B. Hunt, M.A., M.B.Oxon, appointed Assistant Bacteriologist in the Diphtheria Department at the British Institute of Preventive Medicine. C. E. Macguire, M.B., C.M.Aberd., appointed Assistant Medical Officer to the Durham County Asylum. A. M. Martin, M.B., B.S., appointed Assistant Surgeon to the Royal Infirmary, Newcastle-upon-Tyne. L. S. Palmer, M.R.C.S.Eng., L.R.C.P.Lond., appointed Medical Officer to the Reigate and Redhill Cottage Hospital. T. Porter, M.B.Vict., reappointed Assistant Medical Officer to the Manchester Royal Infirmary. A. Brown Ritchie, M.B.Edin., appointed Honorary Surgeon to Hulme Dispensary, Manchester. H. Stallard, B.A.Cantab., M.R.C.S.Eng., L.R.C.P.Lond., appointed House Surgeon to Newark Hospital. C. E. Stephens, M.B , appointed Medical Officer for the First District and the Workhouse of the Morth Witchford Union. J. W. Stoker, L.R.C.P., L.R.C.S., L.M., appointed Medical Officer and Public Vaccinator for the Rainham District of the Romford Union. A. Wilson, F.R.C.S., reappointed Senior Anæsthetist to the Manchester Royal Infirmary.

### ST. THOMAS'S HOSPITAL HOUSE APPOINTMENTS.

The following gentlemen have been selected as house officers from Tuesday, December 4th, 1894: Resident House Physicians, E. A. Saunders, M.A., M.B , B.Ch.Oxon., L.R.C.P., M.R.C.S., and G. G. Genge, L.R.C.P., M.R.C.S. Non-Resident House Physicians, T. G. Nicholson, B.Sc.Lond., L.R.C.P., M.R.C.S. (extension), and A. S. F. Grünbaum, M.A., M.B., B.C.Cantab., L.R.C.P., M.R.C.S. (extension). House Surgeons, H. A. Dickson, L.R.C.P., M.R.C.S. (extension), L. J. Miskin, L.R.C.P., M.R.C.S. (extension), A. W. Cuff, B.A., M.B., B.C.Cantab. (extension), and W. J. C. Merry, M.B., B.Ch.Oxon. (extension). Assistant House Surgeons, A. E. Russell, M.B.Lond., L.R.C.P., M.R.C.S., and H. W. Harding, L.R.C.P., M.R.C.S. Obstetric House Physicians, E. G. E. Arnold, L.R.C P., M.R.C.S. (senior), and W. E. F. Tinley, M.B., B.S.Durh. (junior). Ophthalmic House Surgeons, J. H. Fisher, M.B.Lond., L.R.C.P., M.R.C.S. (senior), and H. G. Toombs, L.R.C.P., M.R.C.S. (junior). Clinical Assistants in the special department for diseases of the throat, A. F. W. King, L.R.C.P., M.R.C.S. (extension), and T. O. Halliwell, L.R.C.P., M.R.C.S. Clinical Assistants in the special department for diseases of the skin, J. W. Laver, L.R.C.P., M.R.C.S. (extension), and H. H. Haward, L.R.C.P., M.R.C.S. Clinical Assistants in the special department for diseases of the ear, L. A. R. Wallace, B.A., M.B.Oxon., L.R.C.P., M.R.C.S., and F. G. Layton, L.R.C.P., M.R.C.S. Clinical Assistant in the electrical department, A. Barry Blacker, M.D., B.S.Durh., L.R.C.P., M.R.C.S.

THE STUDENT OF MEDICINE—*continued.*

### VACANCIES.

The following vacancies are announced this week, the amount of salary in each case being given after the name of the institution:

### Resident Medical Officers.

Birmingham and Midland Eye Hospital.—Assistant House Surgeon. Salary £50 per annum, with apartments and board. Applications to the Chairman of the Medical Board by December 13th.

Birmingham General Dispensary.—Resident Surgeon. Salary £150 per annum. Applications and testimonials to A. Forrest, Secretary, before December 12th.

City of London Hospital for Diseases of the Chest, Victoria Park, E.—House Physician. Appointment for six months. Board, residence, and allowance for washing provided. Applications to the Secretary at the Office, 24, Finsbury Circus, E.C., by December 13th.

Derby County Asylum, Mickleover.—Second Assistant Medical Officer. Salary £100 per annum, increasing £10 annually to £130, with board, lodging, and washing. Applications to the Medical Superintendent.

Manchester Royal Infirmary.—Resident Medical Officer for the Fever Hospital at Monsall; doubly qualified, not less than 25 years of age. Salary £250 per annum, with board and residence. Applications to the Chairman of the Board by December 15th.

Parish of St. Leonard, Shoreditch.—Second Assistant Medical Officer (Male) for the Infirmary, Hoxton Street, N.; doubly qualified. Salary £40 per annum, with rations, furnished apartments, and washing in the Infirmary. Applications to the Medical Officer, 204, Hoxton Street, N.

Royal Berks Hospital, Reading.—House Physician, House Surgeon, and Assistant Medical Officer. Salary £60 per annum each for the first two appointments, with board and lodging. For the third appointment board and lodging will be provided, but no salary. Applications to the Secretary before December 11th.

Salford Union Infirmary, Hope, near Eccles.—Assistant Medical Officer; doubly qualified. Salary £130 per annum, with furnished apartments in the Infirmary. Applications, endorsed Assistant Medical Officer, to T. H. Bagshaw, Clerk to the Guardians, Union Offices, Eccles New Road, Salford, by December 11th.

West London Hospital, Hammersmith Road, W.—House Physician and House Surgeon; tenable for six months. Board and lodging are provided. Applications and testimonials to R. J. Gilbert, Secretary Superintendent, before December 12th.

Royal Ear Hospital, Frith Street, Soho Square.—House Surgeon (six months' appointment). Honorarium 12 guineas.

Sheffield Union.—Junior Assistant Resident Medical Officer to the Workhouse Infirmary, Fir Vale; doubly qualified. Appointment for six months. Salary at the rate of £25 per annum, with board, lodging, and washing. Applications to Joseph Spencer, Clerk to the Guardians, Union Offices, Sheffield, by December 11th.

### Non-Resident Medical Officers.

Hospital for Sick Children, Great Ormond Street, Bloomsbury.—House Surgeon to Out-patients (non-resident). Appointment for six months, but holder will be eligible for a second term of office. Salary 25 guineas. Applications to the Secretary by December 11th.

Owens College, Manchester.—Junior Demonstrator in Anatomy. Annual stipend £100. Applications to the Registrar by December 10th.

West London Hospital, Hammersmith Road, W.—Assistant Anæsthetist (Honorary and Non-Resident). Applications and testimonials to R. J. Gilbert, Secretary Superintendent, before December 12th.

### Medical Appointments.

BIRMINGHAM and MIDLAND EYE HOSPITAL.

WANTED, an ASSISTANT HOUSE SURGEON at the above Hospital, receiving 24,726 patients.

The Assistant House Surgeon must be a Graduate in Surgery of any British University, or a Member, or a Fellow, Licentiate, by examination, of the College of Surgeons of England, Ireland, or Scotland.

Salary £50 per annum with Apartments and Board.

